# How does esophagus look on barium esophagram in pediatric eosinophilic esophagitis?

**DOI:** 10.1007/s00261-016-0712-0

**Published:** 2016-03-24

**Authors:** Abdulrahman Al-Hussaini, Amany AboZeid, Abdul Hai

**Affiliations:** 1 Division of Pediatric Gastroenterology, Children’s Hospital, King Fahad Medical City, P. O. Box 59046, Riyadh, 11525 Kingdom of Saudi Arabia; 2 University of King Saud bin Abdulaziz for Health sciences, Riyadh, Kingdom of Saudi Arabia; 3 Department of Radiology, King Fahad Medical City, Riyadh, 11525 Kingdom of Saudi Arabia

**Keywords:** Eosinophilic esophagitis, Esophageal stricture, Barium esophagram, Narrow-caliber esophagus, Children, Saudi Arabia

## Abstract

**Purpose:**

The clinical, endoscopic, and histologic findings of eosinophilic esophagitis (EoE) are well characterized; however, there have been very limited data regarding the radiologic findings of pediatric EoE. We report on the radiologic findings of pediatric EoE observed on barium esophagram and correlate them with the endoscopic findings.

**Methods and materials:**

We identified children diagnosed with EoE in our center from 2004 to 2015. Two pediatric radiologists met after their independent evaluations of each fluoroscopic study to reach a consensus on each case. Clinical and endoscopic data were collected by retrospective chart review.

**Results:**

Twenty-six pediatric EoE cases (age range 2–13 years; median 7.5 years) had barium esophagram done as part of the diagnostic approach for dysphagia. Thirteen children had abnormal radiologic findings of esophagus (50%): rings formation (*n* = 4), diffuse irregularity of mucosa (*n* = 8), fixed stricture formation (*n* = 3), and narrow-caliber esophagus (*n* = 10). Barium esophagram failed to show one of 10 cases of narrow-caliber esophagus and 10 of 14 cases of rings formation visualized endoscopically. The mean duration of symptoms prior to diagnosis of EoE was longer (3.7 vs. 1.7 year; *p* value 0.019), and the presentation with intermittent food impaction was commoner in the group with abnormal barium esophagram as compared to the group with normal barium esophagram (69% vs. 8%; *p* value 0.04).

**Conclusion:**

Barium swallow study is frequently normal in pediatric EoE. With the exception of narrow-caliber esophagus, our data show poor correlation between radiologic and endoscopic findings.

Eosinophilic esophagitis (EoE) is an allergy-mediated esophageal disease characterized clinically by gastroesophageal reflux disease (GERD)-like symptoms and histologically by eosinophil infiltration of esophageal mucosa by >15 eosinophils/high power field (HPF) [1]. The clinical, endoscopic, and histologic findings of EoE are well characterized, however, there have been limited data regarding the radiologic findings of EoE in children. Because dysphagia is a common symptom of EoE and is not specific for the disorder, a diagnostic work-up that includes barium esophagram is frequently performed to exclude obstructive etiologies.

The radiologic manifestations of EoE have been reported in adult case reports and series [2–4]. Few reports described the fluoroscopic findings in pediatric EoE [5–9]. These reports have described normal fluoroscopic study in 52% to 70% and infrequent occurrence of esophageal strictures, rings, and mucosal irregularity. The relatively long mean interval between the barium studies and endoscopy and non-blindness of radiologist to clinical and endoscopic data were important limitations of these reports. In the study by Binkovitz et al. [5], there was a median of 7.5 days (but mean of 48.4 days) between the barium study and endoscopy, while in the study by Diniz et al. [6], the majority of fluoroscopic studies (104/112; 93%) were performed before esophageal biopsy (median 40 days before biopsy; range 85 months before biopsy to 1 month after biopsy). As a result, it can be argued that disease activity might have changed in the interval between these procedures, and that radiologic findings may not have directly correlated with the endoscopic findings obtained weeks or months before or after the fluoroscopic study.

In this retrospective study, we report the radiologic findings of EoE on barium swallow study performed within 1 week prior to upper endoscopy and correlate them with the endoscopic findings.

## Methods and materials

We identified children diagnosed with EoE in our center from April 2004 to December 2015. EoE was defined as esophageal mucosal infiltration with a peak eosinophil count ≥15 eosinophils/high-powered field in biopsies obtained from multiple levels of esophagus. The hospital PACS was then used to locate barium esophagram performed in these patients. EoE cases who underwent barium esophagram within 1 week prior to upper endoscopy, were included in the study. Clinical records, laboratory data, and pathology reports were reviewed. Endoscopy reports were also reviewed for the presence or absence of a small-caliber esophagus, ringed esophagus, mucosal furrowing, white exudates, or strictures and compared with the radiological findings.

Two pediatric radiologists, with more than 10 years’ experience, independently reviewed each fluoroscopic study for esophageal strictures, narrow-caliber esophagus, rings formation, and mucosal irregularity. Both radiologists were blind to clinical and endoscopic information. The radiologists met after their independent evaluations to reach a consensus on each case. Narrow-caliber esophagus was defined as either short-segment narrow caliber if the stenosis was limited to one-third of esophagus or long-segment narrow caliber if the stenosis involved more than one-third of esophagus. The term “esophageal stricture” was used to describe a very short distinct stenosis. If a stricture or narrow-caliber esophagus was present, its location within the esophagus was recorded as occurring in the proximal (cervical to T2 level), mid (T3–T6), or lower (T7 to thoracolumbar junction) esophagus. The severity of stenosis was graded into three groups as follows: “low-grade stenosis” that allows passage of standard pediatric upper endoscope (outer diameter 8.6 mm) with little resistance; “intermediate-grade stenosis” that allows passage of the neonatal endoscope (outer diameter 6 mm) but not of a standard upper endoscope; and “high-grade stenosis” that does not allow passage of a 5.9-mm neonatal endoscope.

All of the barium swallow studies were performed using a single-contrast low-density barium suspension (Entrobar) and included prone, right anterior oblique views. All studies were performed using digital fluoroscopic equipment (Diagnostic 76, Philips, Eindhoven, The Netherlands; or Sireskop SD, Siemens, Erlangen, Germany). The clinical records and laboratory data were reviewed and the endoscopic findings were compared with the radiographic findings. This retrospective study was performed with institutional review board approval.

### Statistical analysis

Data were analyzed using SPSS PC+ version 21.0 statistical software. Descriptive statistics (mean, standard deviation, and percentages) were used to describe the quantitative and categorical study variables. Student’s *t* test for independent samples was used to compare the mean values of quantitative variables. Fisher’s exact test was used to observe an association between categorical study variables and outcome variable (Abnormal barium esophagram). A *p* value of <0.05 was considered as statistically significant.

## Results

During the study period, 50 pediatric EoE cases were diagnosed (age range 1–14 years, median 8 years; 36 males). Twenty-six cases (age range 2–13 years, median 7.5 years; 19 males) had barium swallow study done within 1 week prior to upper endoscopy, as part of the diagnostic approach. The details of clinical, radiologic, and endoscopic findings of the 26 patients are shown in Table 1. The main indication for the barium studies were dysphagia and vomiting. Thirteen patients had normal barium esophagram (50%); the remaining 13 patients had abnormal fluoroscopic findings: rings formation in 4 (15%), narrow-caliber esophagus in 10 (38.5%), esophageal stricture in 3 (11.5%), and irregularity of esophageal contour in 8 patients (31%).

**Table 1:** Summary of the clinical, radiologic, and endoscopic findings of the 26 pediatric patients with EoE

Patient	Age (years)	Sex	Symptoms	Duration of symptoms (years)	Barium swallow findings	Endoscopic findings
1	8	Male	Dysphagia, weight loss	1	Rings in UE	Rings, mucosal furrowing
2	9	Female	Dysphagia, intermittent food impaction	3	Normal	“Low-grade” short narrow caliber in ME (4 cm), rings, mucosal furrowing
3	2	FemaleF	Feeding difficulty vomiting, FTT	1	Normal	Mucosal furrowing
4	4	Female	Feeding difficulty vomiting, FTT	0.6	Stricture at cervical region of esophagus, short narrow caliber in UE, mucosal irregularity	Stricture at 2 cm below UES, “intermediate-grade” short narrow caliber in UE (3 cm), white exudates, mucosal furrowing
5	6	Male	Dysphagia	2	Rings in ME, mucosal irregularity in ME and LE	Rings, mucosal furrowing
6	4	Male	Dysphagia	1	Short-segment narrow caliber in LE, mucosal irregularity	“intermediate-grade” short-segment narrow caliber in LE (3 cm), mucosal furrowing
7	10	Male	Dysphagia, weight loss	3	Stricture at cervical region of esophagus, long-segment narrow caliber in UE and ME, rings in UE, mucosal irregularity in UE	“High-grade” long-segment narrow caliber in UE and ME (8 cm), rings, white exudates, mucosal furrowing
8	8	Male	Dysphagia, weight loss	1	Normal	Rings, white exudates, mucosal furrowing
9	5	Male	Dysphagia	0.8	Normal	Mucosal furrowing
10	4	Male	Dysphagia, vomiting	2	Normal	Mucosal furrowing
11	5	F	Dysphagia	0.4	Normal	Mucosal furrowing
12	4	Male	Dysphagia	2	Normal	Mucosal furrowing, rings, white exudates
13	11	Female	Dysphagia, intermittent food impaction	3	Normal	Mucosal furrowing, rings, white exudates
14	5	Female	Dysphagia, vomiting	2	Normal	Mucosal furrowing, white exudates
15	10	Male	Dysphagia, intermittent food impaction	2	Stricture at 3 cm from UES, long-segment narrow caliber in ME and LE	Stricture at 3 cm from UES, “high-grade” long-segment narrow caliber at UE and ME (14 cm), rings, white exudates, mucosal furrowing
16	12	Female	Dysphagia	2	Rings	Rings, mucosal furrowing
17	8	Male	Dysphagia, intermittent food impaction	7	Short-segment narrow caliber in ME	“Intermediate-grade” short-segment narrow caliber (3 cm) in ME, rings, white exudates, mucosal furrowing
18	13	Male	Dysphagia, heart burn	2	Normal	Normal
19	12	Male	Dysphagia, intermittent food impaction	7	Long-segment narrow caliber in ME and LE, Rings	“Intermediate-grade” long-segment narrow caliber (8 cm), rings, white exudates, mucosal furrowing
20	7	Male	Dysphagia, intermittent food impaction, weight loss	2	Long-segment narrow caliber in UE and ME, mucosal irregularity	“Intermediate-grade” long-segment narrow caliber in UE and ME (14 cm), rings, white exudates, mucosal furrowing
21	9	Male	Dysphagia, food impaction, weight loss	5	Narrow caliber at UE, mucosal irregularity	“Intermediate-grade” short narrow caliber at UE (4 cm), white exudates, rings, mucosal furrowing
22	10	Male	Dysphagia, food impaction	7	Narrow short-segment caliber in ME, mucosal irregularity	“High-grade” short narrow caliber at ME (4 cm), white exudates, rings, mucosal furrowing
23	3.5	Male	Vomiting	3	Normal	Mucosal furrowing
24	2.2	Male	Vomiting dysphagia	1.5	Normal	Mucosal furrowing
25	8.5	Male	Dysphagia, food impaction	1	Normal	White exudates, mucosal furrowing
26	7	Male	Dysphagia, food impaction, weight loss	6	Long-segment narrow caliber throughout the entire course of esophagus	“Low-grade” long narrow caliber (20 cm), white exudates, mucosal furrowing, mucosal ulcer

F, female; FTT, failure to thrive; LE, lower esophagus; M, male; MU, mid-esophagus; UE, upper esophagus; UES, upper-esophageal sphincter

**Fig. 1:**
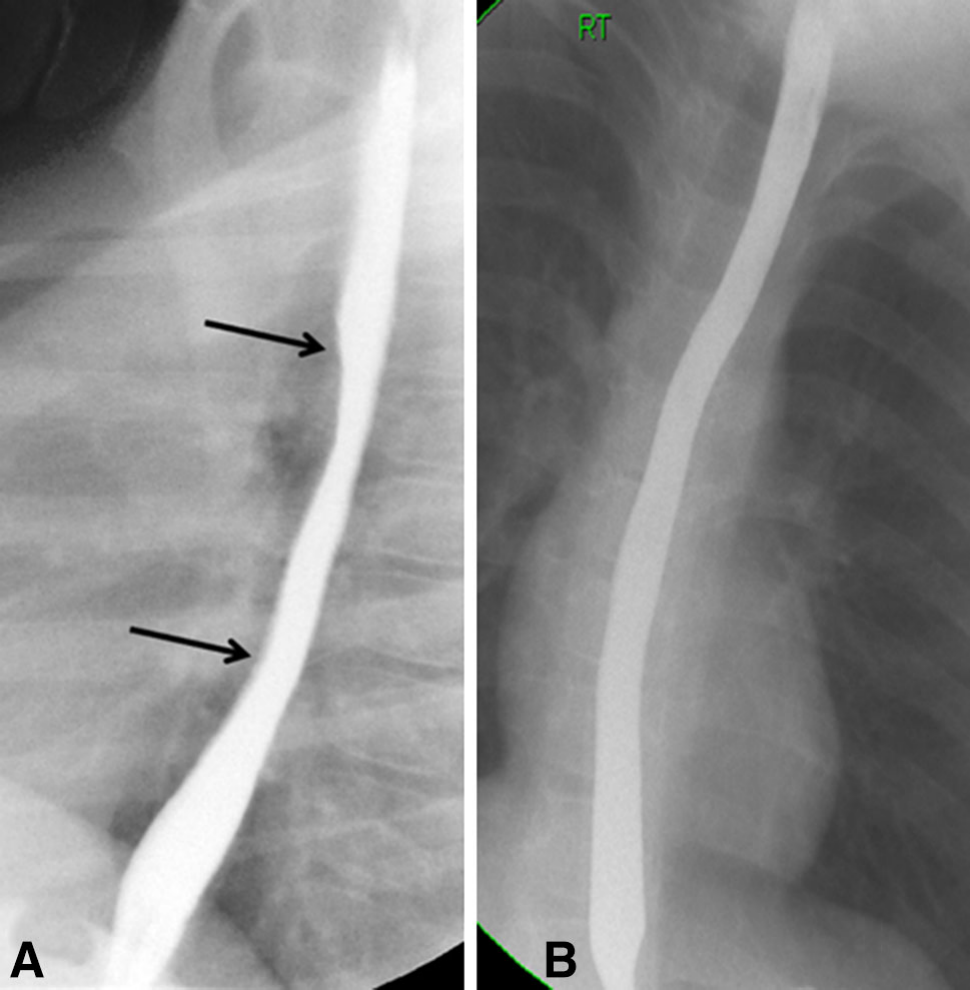
**A** Lateral views a barium esophagogram in patient 17 shows a short-segment narrow-caliber esophagus involving mid-esophagus (between the two *arrows*). **B** Anteroposterior view for a barium esophagogram in patient 26 shows long-segment narrow-caliber throughout the entire course of esophagus

**Fig. 2:**
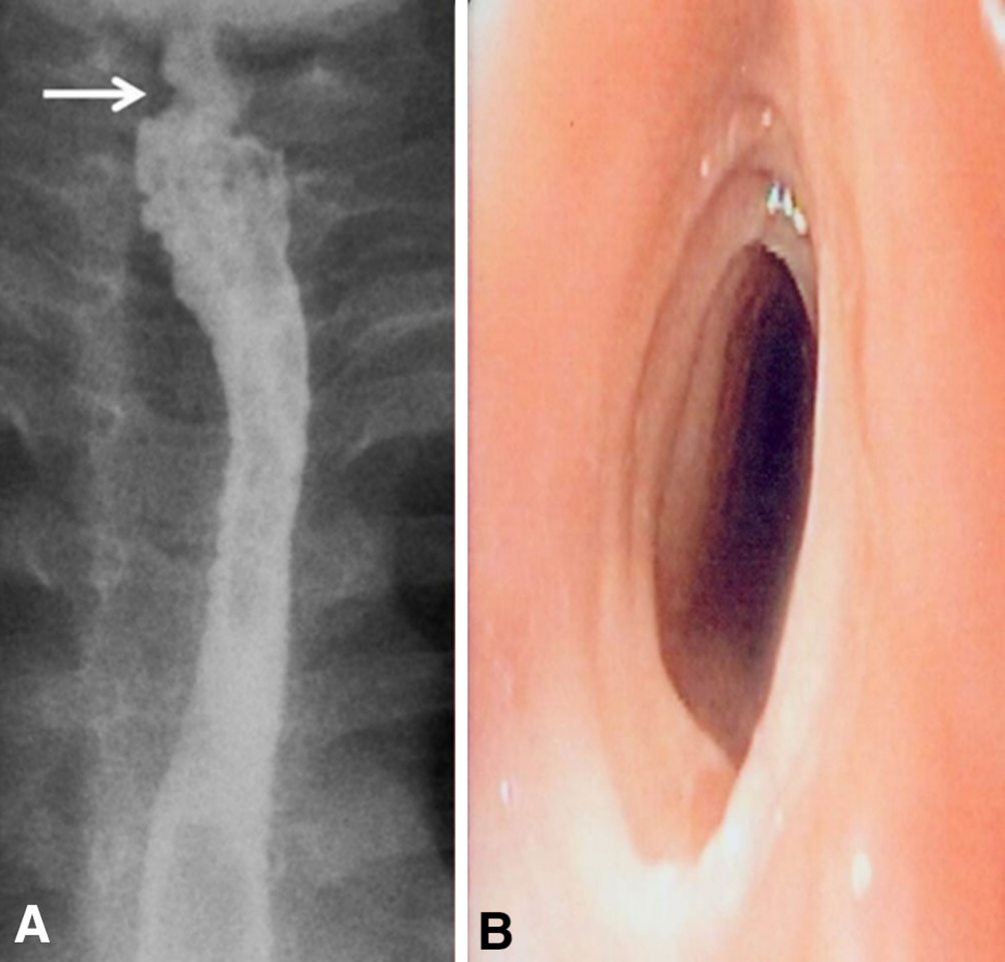
**A** Barium esophagogram in patient 7 shows a focal stricture at 2 cm below upper esophageal sphincter (*upper arrow*), a long-segment narrow-caliber esophagus distal to the stricture involving both upper esophagus and mid-esophagus, and mucosal irregularity. **B** Endoscopic view for the focal stricture in the same patient. It demonstrates a multiple, fixed, closely spaced, concentric rings traversing the stricture

Barium esophagram failed to show one of the 11 cases of narrow-caliber esophagus visualized endoscopically. Patient 2 had esophageal narrowing unrecognized on barium esophagogram and a subtle “low-grade” narrow-caliber esophagus was noticed during endoscopy. Six had short-segment narrow-caliber esophagus (three in mid-esophagus, two in upper esophagus, and one in lower esophagus), and five had long-segment narrow-caliber esophagus (three in upper and lower esophagus, one in mid-esophagus and lower esophagus, and one along the entire esophagus) [median length of the narrowing was 4 cm, range 3–20 cm] (Fig. 1). The esophageal narrowing was “high-grade stenosis” in three patients, “intermediate-grade stenosis” in six, and “low-grade stenosis” in two. Three cases (patients 4, 7, and 15) with narrow-caliber esophagus also had esophageal stricture 2–3 cm below the upper esophageal sphincter (Fig. 2) while in seven cases, barium esophagram and endoscopy demonstrated a uniformly narrow esophageal lumen. Real-time evaluation during fluoroscopic examination showed lack of distensibility of the esophageal lumen and the presence of a static narrowed caliber of the esophagus. During upper endoscopy, there was diffuse esophageal narrowing and the esophageal lumen looked like a rigid pipe non-compliant to insufflation of air.

**Fig. 3:**
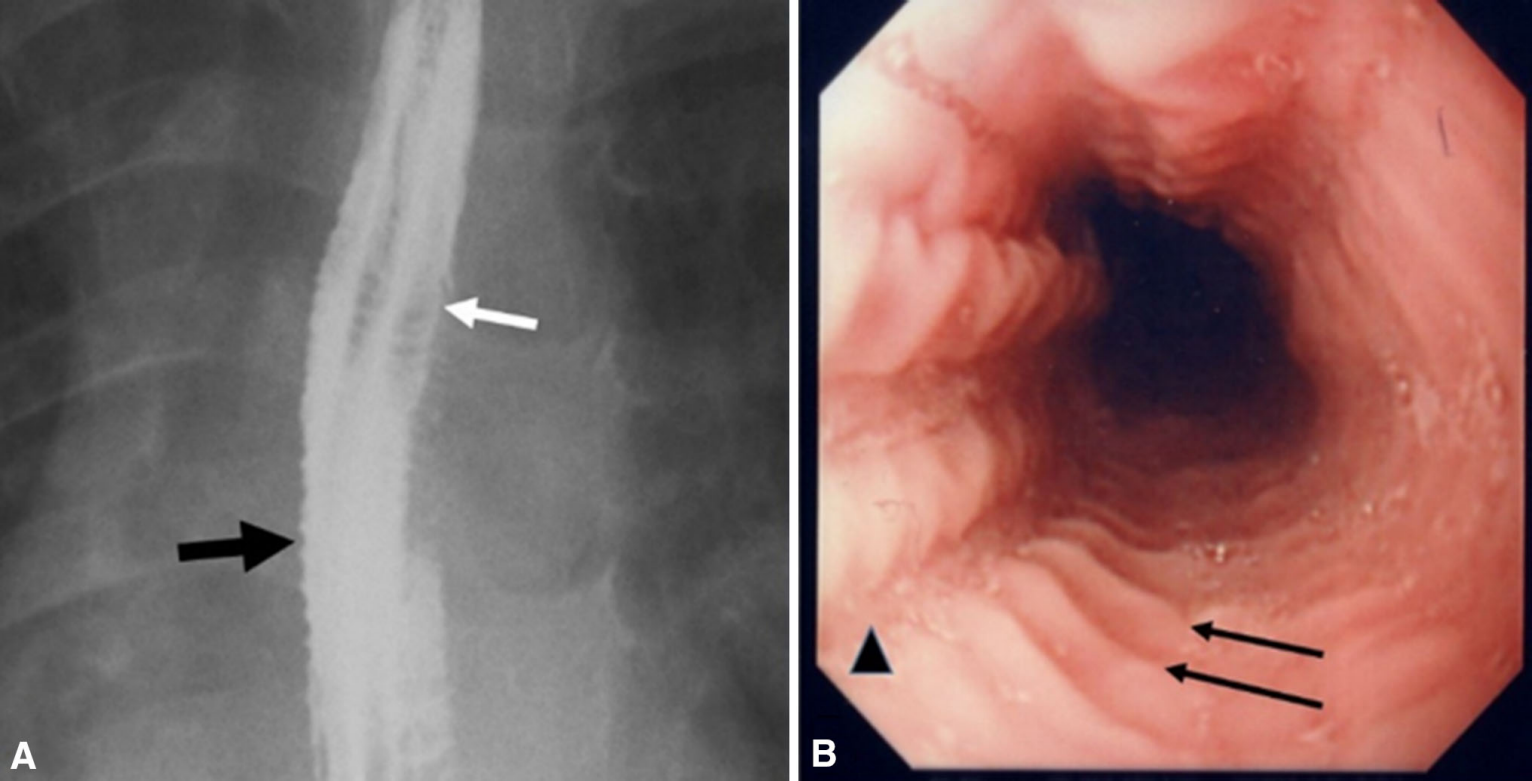
**A** Esophagram in patient 5 with history of recurrent food impactions and dysphagia shows multiple esophageal rings (*white arrow*), giving the appearance of a corrugated or ringed esophagus, and mucosal irregularity (*black arrow*). **B** Endoscopy showed multiple transverse rings (*arrows*) and mucosal furrowing (*arrow head*)

Concentric ring formation was found in four patients (Patients 1, 5, 7, and 19) on barium esophagram, appearing as multiple closely spaced, concentric rings (Fig. 3A) over the upper two-thirds of esophagus in three patients and over the lower one-third in one patient. The endoscopic equivalent of these rings was documented as persistent finding in the four patients (Fig. 3B). However, barium esophagram failed to show rings formation visualized on upper endoscopy in another ten patients. The rings in these ten cases were seen as a transient endoscopic finding, better visualized during peristaltic contractions of esophagus.

Table 2 shows the comparison of the group of 13 EoE patients with abnormal barium study and the group of 13 EoE patients with normal barium study. The mean duration of symptoms prior to diagnosis of EoE was longer (3.7 vs. 1.7 year; *p* value 0.019) and the presentation with intermittent food impaction was commoner in the group with abnormal barium esophagram as compared to the group with normal barium esophagram (69% vs. 8%; *p* value 0.04). Other clinical and endoscopic variables were not statistically significantly different between both groups.

**Table 2:** Comparison of eosinophilic esophagitis patients with normal and abnormal barium study

Variables	Abnormal barium study (*n* = 13)	Normal barium study (*n* = 13)	*p* value
Age [mean (SD)]	8 (2.5)	6.4 (3.9)	0.39
Duration of symptoms [mean (SD)]	3.7 (2.3)	1.7 (0.8)	0.019
Gender (males, %)	11 (85)	8 (61.5)	0.17
Dysphagia (%)	13 (100)	11 (85)	0.45
Vomiting (%)	1 (8)	6 (46)	0.10
Heartburn (%)	0 (0)	1 (10)	0.45
Weight loss (%)	9 (69%)	2 (15)	0.045
Food impaction (%)	8 (69%)	1 (8%)	0.04
Asthma (%)	7 (58.3)	3 (30)	0.20
Eczema (%)	3 (23)	2 (15.4)	0.44
Family history of atopy (%)	8 (69)	9 (84.6)	0.39
Eosinophilia (%)	7 (54)	7 (62)	0.9

**Fig. 4:**
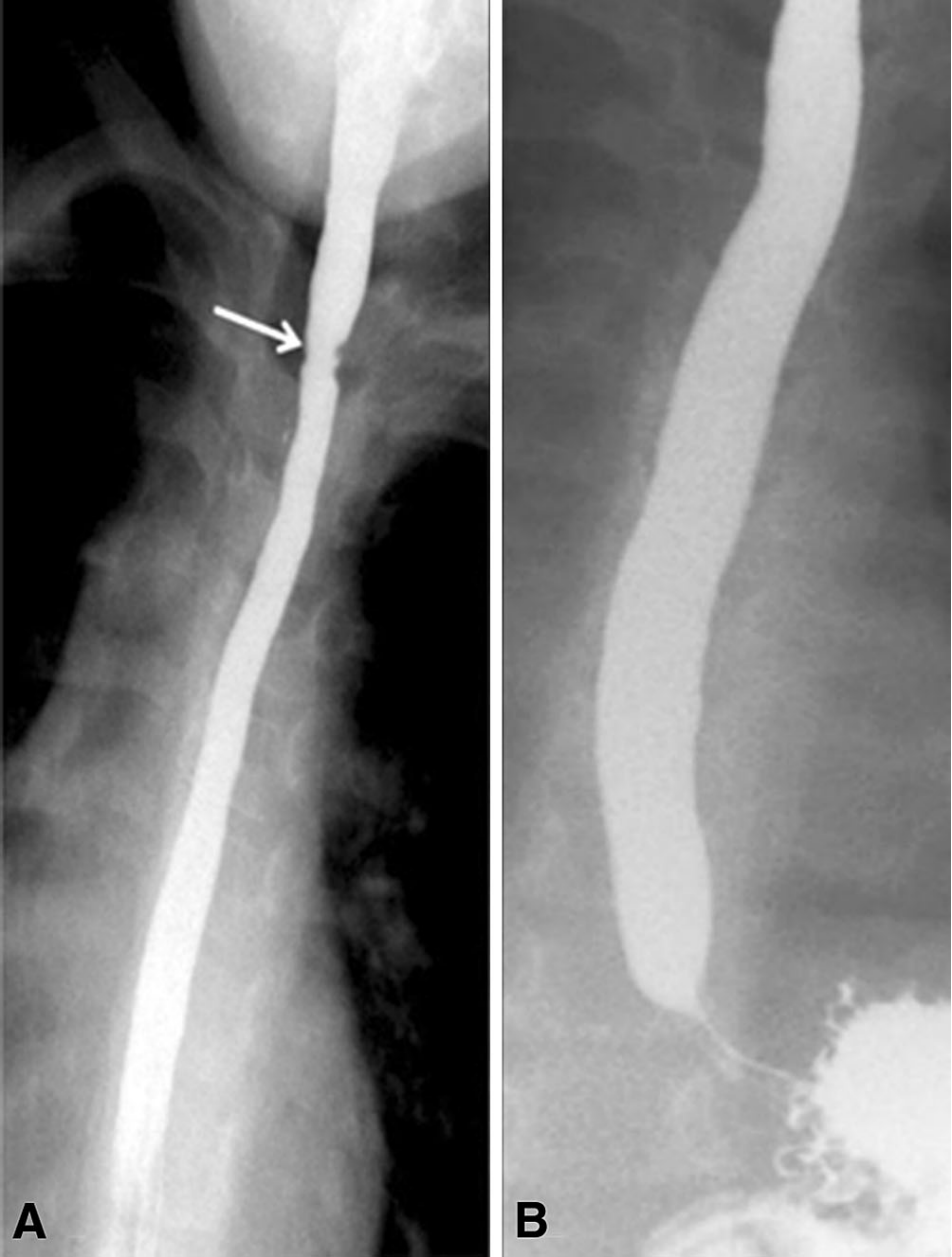
**A** Barium esophagogram in patient 15 shows a stricture at 3 cm below upper esophageal sphincter, long-segment narrow caliber in middle and lower esophagus. **B** Barium esophagogram in the same patient after three sessions of dilation (up to size 14 mm)

Ten children with narrow-caliber esophagus underwent endoscopic dilation using Savary dilators that led to good response (Fig. 4), while patient 26 responded dramatically to a 3-month course of swallowed inhaled fluticasone. All the 26 patients received swallowed aerosolized fluticasone propionate from a metered dose inhaler at a dose of 250 micrograms twice daily for children <10 years of age and 500 mcg twice daily for children >10 years of age.

This dose was dispensed for 2 months and tapered to a maintenance dose of 125 micrograms twice daily.

## Discussion

The most important finding of our study is the poor sensitivity of barium esophagram to diagnose EoE as 50% of our patients had normal fluoroscopic findings. Therefore, in order to be diagnosed with EoE, patient must undergo upper endoscopy and multiple level biopsies from esophagus to fulfill histopathological criterion (≥15 eosinophil/HPF). Another important finding is the lack of good correlation between radiologic and endoscopic findings, with the exception for esophageal stricture and narrow-caliber esophagus. These two findings speak against the routine use of esophageal fluoroscopy as a routine diagnostic test for EoE, but it can be helpful in selected cases to characterize anatomic abnormalities that can be difficult to define endoscopically and to provide information on the length and diameter of esophageal narrowing.

EoE represents a chronic, immune/antigen-mediated esophageal disease characterized clinically by symptoms related to esophageal dysfunction and histologically by eosinophil-predominant inflammation with infiltration of esophageal mucosa by >15 eosinophils/HPF [1]. The cause of EoE is not well understood, but food allergy has been implicated, especially in light of positive treatment trials with elemental and elimination diets [10–12]. Majority of the patients with EoE in our series had an allergic background, illustrated by peripheral eosinophilia and high prevalence of atopy disease in patients and relatives, supporting several studies that have demonstrated that EoE is closely related to atopy [12, 13]. Due to the similar clinical presentations of GERD and EoE in children, the distinction between these two entities can be difficult. Therefore, the diagnosis of EoE may be overlooked for several years before their gastrointestinal symptoms grow severe enough that parents seek medical advice due to increasing swallowing difficulty or recurrent food impactions. At endoscopy, EoE is characterized by longitudinal mucosal furrowing, white exudates, rings formation, mucosal shearing, esophageal stricture, and esophageal rings [5]. Although these findings are typical of EoE, however, none of them is pathognomonic. Similarly, the esophageal radiologic findings observed in our study and others [5, 6], like mucosal irregularity, stricture, and rings formation are not diagnostic of EoE as they have been observed in patients with GERD. Although small-caliber esophagus is more specific for diagnosis of EoE, this radiologic finding is uncommon and thus not sensitive for the diagnosis of EoE. GERD typically involves the distal esophagus; therefore the presence of one or more of the above mentioned radiologic findings in proximal esophagus is strongly suggestive of EoE.

Two pediatric studies have described the fluoroscopic findings of EoE [5, 6]; 52% to 70% of the patients had no fluoroscopic findings, similar to our study (50%). The imaging findings of EoE can vary depending on the severity eosinophilic infiltration and the degree of remodeling of esophageal wall. The esophagus in EoE passes through two phases, an “inflammatory phase” characterized by extensive eosinophilic infiltrates of esophageal mucosa, that can progress, if untreated, into a “fibrotic phase” characterized by sub-epithelial fibrosis and subsequent thickening. Results of high-resolution endoscopic ultrasonography have shown that patients with EoE and esophageal rings, stricture, and narrow-caliber esophagus have diffuse esophageal wall thickening [14], caused by scarring and fibrosis [15, 16]. Therefore, one possible explanation of the low frequency of abnormal radiologic findings in pediatric EoE is that abnormal fluoroscopic findings occur in advanced EoE when the deeper layers of the esophagus (submucosa and muscular layer) are involved with fibrosis and full-wall thickening. This might explain that the most frequent abnormal radiologic findings in our study were those associated with fibrosis, i.e., esophageal rings, stricture, and narrow-caliber esophagus. EoE limited to superficial mucosal layer, i.e., “inflammatory phase,” therefore may not be associated with abnormal radiologic findings. Data from our study indicate that the clinical phenotype of EoE, that could predict the presence of abnormal barium esophagram, particularly esophageal narrowing, was characterized by longer duration of symptoms prior to diagnosis and food impaction and weight loss on presentation.

All the 26 patients in our cohort and majority of patients in the other two pediatric studies [5, 6] presented with dysphagia but anatomical abnormalities could be identified in less than 40% of the cases. The cause of the dysphagia in these children is felt to be due to dysmotility rather than a fixed narrowing of the esophageal lumen [17]. A narrow-caliber esophagus was not recognized by the radiologists in one patient. Therefore, another possible explanation of the low frequency of abnormal radiologic findings in EoE is that findings of a narrow-caliber esophagus or proximal cervical esophageal stricture might be overlooked. Narrow-caliber esophagus is a subtle finding that can be more difficult to recognize on barium study images than a short focal esophageal stricture because of their long length, uniform luminal diameter, and smooth contour without abrupt transition from normal to small caliber. Therefore, communication with radiologist regarding the indication for barium esophagram study is important so that the entire esophagus, including the caliber, esophageal motility, and distensibility of the esophageal lumen, will be fully assessed. In our series, the rings were more easily recognized at endoscopy than at barium study (only 4 of 14 patients with rings at endoscopy had rings at barium study); the transient nature of the rings finding on endoscopy may have contributed to the negative finding on barium study.

There are several limitations in our study. Our study had the inherent limitations of a retrospective study, including interpretation bias, as the radiologists who reviewed the images were aware that the barium studies belong to EoE patients. There is no standard reference for the normal esophageal diameter in children. Therefore, the diagnosis of a small-caliber esophagus in our study was made through a subjective interpretation of the caliber of the esophagus, but was confirmed by endoscopy. It is the standard practice to perform a single-contrast esophagram on children in our hospital and mucosal irregularity is difficult to identify when performing a single-contrast upper GI examination. There is a likelihood of selection bias in our case series that probably resulted from the selection of patients with severe dysphagia and intermittent food impaction for radiologic evaluation prior to upper endoscopy. On the other hand, one strong point in our study is that endoscopy was performed within 1 week of the barium studies in all patients, as compared to the long mean interval between the barium studies and endoscopy in the two pediatric series in the literature [5, 6]. In addition, the endoscopy procedures in our study being performed by a single pediatric endoscopist has standardized methodology of describing the endoscopic findings and characterization of esophageal narrowing, thus eliminating operator bias. These advantages in our study increase the likelihood that the endoscopic findings reflect the radiologic findings.

In conclusion, the sensitivity of barium esophagram as a diagnostic test for EoE appears to be low. With the exception of narrow-caliber esophagus, our data show poor correlation between radiologic and endoscopic findings. Therefore, barium esophagram cannot be recommended as a routine diagnostic test for EoE but it can be useful in selected cases to characterize anatomic abnormalities particularly cases characterized by long duration of symptoms and presentation with recurrent episodes of food impaction.
